# Associations between HLA and autoimmune neurological diseases with autoantibodies

**DOI:** 10.1186/s13317-019-0124-6

**Published:** 2020-01-22

**Authors:** Sergio Muñiz-Castrillo, Alberto Vogrig, Jérôme Honnorat

**Affiliations:** 1 French Reference Center on Paraneoplastic Neurological Syndromes and Autoimmune Encephalitis, Hospices Civils de Lyon, Hôpital Neurologique, 59 Boulevard Pinel, 69677 Bron Cedex, France; 2 SynatAc Team, Institut NeuroMyoGène, INSERM U1217/CNRS UMR 5310, Université de Lyon, Université Claude Bernard Lyon 1, Lyon, France

**Keywords:** HLA, Autoantibodies, Myasthenia gravis, Autoimmune encephalitis, Limbic encephalitis, Paraneoplastic neurological syndrome, Neuromyelitis optica

## Abstract

Recently, several autoimmune neurological diseases have been defined by the presence of autoantibodies against different antigens of the nervous system. These autoantibodies have been demonstrated to be specific and useful biomarkers, and most of them are also pathogenic. These aspects have increased the value of autoantibodies in neurological practice, as they enable to establish more accurate diagnosis and to better understand the underlying mechanisms of the autoimmune neurological diseases when they are compared to those lacking them. Nevertheless, the exact mechanisms leading to the autoimmune response are still obscure. Genetic predisposition is likely to play a role in autoimmunity, HLA being the most reported genetic factor. Herein, we review the current knowledge about associations between HLA and autoimmune neurological diseases with autoantibodies. We report the main alleles and haplotypes, and discuss the clinical and pathogenic implications of these findings.

## Background

Autoimmune neurology is an expanding field that has seen a huge development in recent years. Most of this progress is due to the discovery and characterization of autoantibodies (Ab) directed against antigens of the peripheral and/or central nervous system, and which are used as biomarkers of these diseases. Some of these Ab have allowed to better define already known entities, such as Ab against aquoporin-4 (anti-AQP4 Ab) in neuromyelitis optica (NMO) [1, 2]. In contrast, the identification of Ab against *N*-methyl-d-aspartate receptor (anti-NMDAR Ab) and other Ab related to autoimmune encephalitis has led to the description of completely new disorders that have revolutionized neurological practice [3]. Moreover, most of these Ab are thought to be pathogenic [4, 5], although the primary trigger of the abnormal immune response is still unknown for the majority of these diseases.

Genetics is likely to play an important role in the pathogenesis of Ab-associated autoimmune neurological diseases. First, it is not uncommon that patients or their relatives present with other systemic autoimmune diseases, suggesting a shared predisposition to self-tolerance loss. Second, some acquired factors (such as cancer in paraneoplastic neurological syndromes, PNS) may lead to the development of an autoimmune disease in some subjects, while not in others, probably reflecting the interaction with a high-risk genotype.

Human leukocyte antigen (HLA) is the main genetic factor related to autoimmune diseases, accounting for a half of known genetic predisposition [6]. Although more than 200 associations between HLA and disease (immune-mediated or not) have been described, the underlying pathogenic mechanisms remain poorly defined [7–9]. Initially, the particular genetic characteristics of HLA, and the complex interaction with other genes and environment have prevented further clinically-meaningful developments in this field [6, 8, 9]. However, in recent years technological advances and increasing knowledge about peptide-HLA interactions has enabled to further understand the role of HLA in disease susceptibility [8, 9].

In this review, we summarize the current knowledge about associations between HLA and autoimmune neurological diseases with Ab used as biomarkers, describing the main reported alleles and haplotypes, and providing details of some clinical and pathogenic aspects. The review focuses on PNS, autoimmune encephalitis, myasthenic syndromes and NMO, as in these diseases an antigen is clearly identified suggesting specific mechanisms.

## HLA genetics, structure, and function

The HLA-complex is located on chromosome 6p and represents the most dense and polymorphic region of the human genome [7, 10, 11]. HLA haplotypes (alleles from different loci on the same chromosome) are made up of relatively fixed allele combinations because HLA displays the highest degree of linkage disequilibrium (LD) in the genome, meaning that alleles from closely located loci are found in a non-random distribution [7, 8, 11, 12]. LD sometimes makes it difficult to establish the causal locus in disease-association studies, but conversely it allows to infer HLA by imputation methods in large samples and population studies [8, 12].

**Fig. 1:**
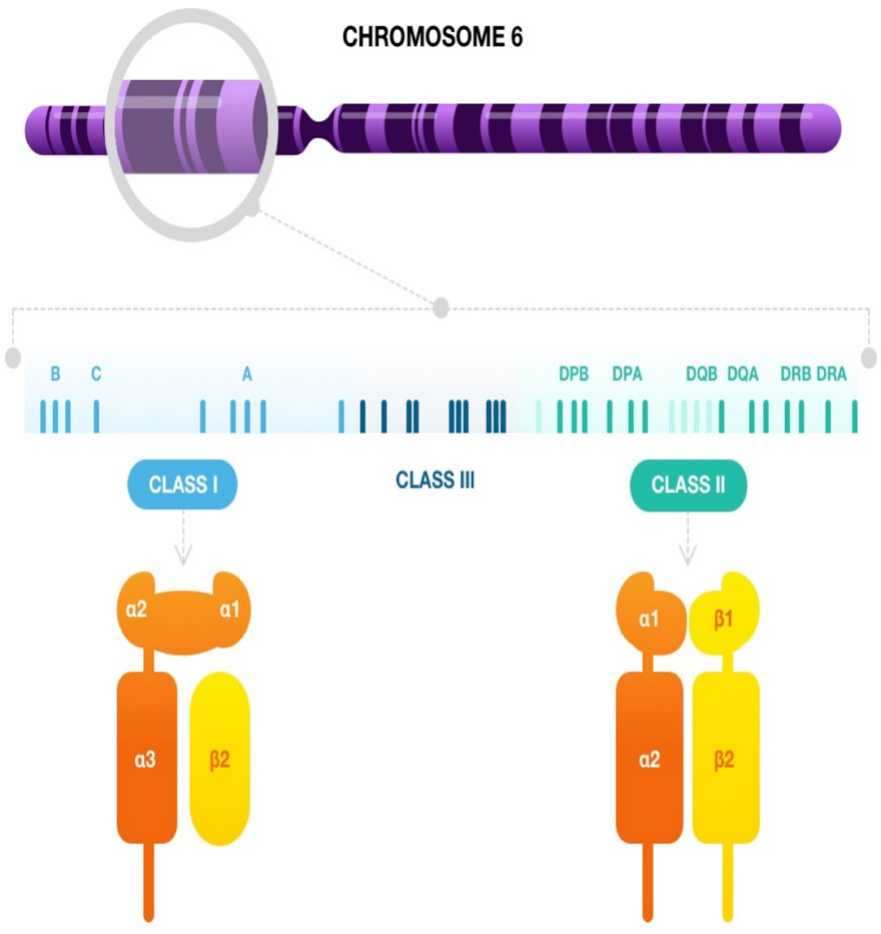
HLA-complex on chromosome 6, showing the main class I and II HLA genes (top). Structure of class I (bottom left) and class II (bottom right) HLA molecules. The alpha chain of class I molecule has three domains (α1–3) and includes the peptide-binding groove. The β2-microglobulin (β2) is not encoded by HLA genes. The class II molecules are made up of one alpha and one beta chain that each contains two domains (α1, α2, β1, β2); both form the peptide-binding groove

The HLA complex is divided in three regions: class I, II and III (Fig. 1) [10, 11]. Class III differs from the others as it only contains non-HLA genes and many of them are even unrelated to the immune system [11]. Classic class I HLA includes HLA-A, B, and C; the main class II HLA genes are DP, DQ, and DR [10, 11]. Class I genes encode the alpha chain of the class I HLA molecule, which includes the peptide-binding groove; the beta-chain is the beta2-microglobulin, which is encoded by a gene that is placed outside the HLA-complex on chromosome 15 [10, 11]. Class II HLA genes code for alpha (for example, DPA) or beta (i.e. DPB) chains; both of them form the peptide-binding groove [10, 11]. HLA alleles follow a nomenclature based on different digit-levels [12]. The first two digits represent the allele group, which usually corresponds to the serological antigen. The two following digits describe the allelic subtype that code for different proteins. Further digits are used to distinguish DNA variants (synonymous substitutions in exons or polymorphisms in introns) that do not change the amino acid sequence. Most of the variability of HLA concentrates in the exons that code the amino acids of the peptide-binding groove [9, 11, 12].

Class I HLA genes are expressed by all nucleated cells. They present peptides derived from intracellular proteins to CD8 T-cells, but they can also be recognized by NK-cells [10–12]. Conversely, class II HLA is only expressed by professional antigen presenting cells that interact with CD4 T-cells, and this class presents peptides derived from extracellular proteins [10–12]. The HLA peptide-binding groove has two walls and a floor [9, 10]. The T cell receptor (TCR) must be placed diagonally to interact correctly with HLA. The less polymorphic loops within the variable domain of TCR, CDR1 and CDR2, bind the walls. Thus, the peptide is located between the floor of the peptide-binding groove and the highly variable loop of the TCR (CDR3) [10]. The amino acids at the ends of the peptide interact with “pockets” in the floor (some of them being decisive to establish peptide specificity of the HLA molecule), whereas the central part of the peptide protrudes out of the groove and interacts with CDR3 [9, 10]. The process also needs the involvement of the co-receptors CD4 and CD8 [10].

HLA also plays a major role in T-cell selection in the thymus. During the first step, only the T-cells whose TCR binds to HLA molecules expressed by thymic cortex epithelial cells are positively selected, while the others are eliminated by apoptosis [10]. Immature thymocytes express both CD4 and CD8, but in case their TCR recognizes preferentially class I HLA, they will downregulate CD4 and upregulate CD8; the opposite occurs with class II HLA [10]. T-cells then move to the thymic medulla, where a large amount of self-peptides are exposed bound to HLA I and II. In this phase, thymocytes that establish high-affinity interactions are eliminated in order to suppress auto-reactivity and promote self-tolerance [10]. Many of the mechanisms proposed to be involved in the association of HLA with disease imply escape of thymic negative selection because of disturbances in TCR-peptide-HLA interaction [9]. In addition, altered gene expression, epistatic interactions with genes outside the HLA-complex, and modification of the peptide binding repertoire by small exogenous molecules such as drugs, have been also described [7–9].

## HLA and autoimmune diseases

Several associations between HLA and different autoimmune diseases have been reported [7]. Most of them belong to class II HLA, such as those described in type 1 diabetes mellitus, in which DQA1*05:01-DQB1*02:01-DRB1*03:01 (DQ2-DR3) and DQA1*03:01-DQB1*03:02-DRB1*04 (DQ3-DR4) represent the two haplotypes conferring the highest risk, but many other haplotypes of intermediate risk or even with protective effects are known [7, 11]. In addition, evidence exists regarding the potential effects of HLA-mediated antigen presentation in the pathogenesis of type 1 diabetes mellitus [9]. Other well-known class II HLA associations in which HLA-mediated antigen presentation may be involved are DRB1*15 in multiple sclerosis, DRB1*04 in rheumatoid arthritis, and DQB1*02 and DQB1*03 (DQ8) in celiac disease [7, 9, 11]. In contrast, class I HLA associations are much more uncommon; the most characterized being HLA-B*27 with ankylosing spondylitis [7, 11].

## Autoimmune neurological diseases with autoantibodies

In the following sections, we will review the main HLA alleles and haplotypes that were found to be significantly more frequent (after an appropriate adjustment for multiple comparisons) in neurological diseases with Ab, and the importance to their pathogeneses and clinical aspects. Nevertheless, because of the scarcity and also sometimes the recent description of many of these diseases, samples are usually small and results have been not reproduced in different populations or by distinct research groups. However, some findings are promising as they enable us to further understand the underlying mechanisms, or even constitute good biomarkers of these diseases.

### Paraneoplastic neurological syndromes with anti-Hu autoantibodies

The most common onconeural Ab related to PNS is anti-Hu Ab [13]. Its clinical spectrum is very wide, ranging from peripheral manifestations (e.g. sensory neuronopathy) to central syndromes (e.g. limbic encephalitis). Also, multifocal involvement in the form of encephalomyelitis is not rare [14]. Anti-Hu Ab react against RNA-binding proteins located in the nucleus, and they are not believed to be pathogenic [15]. Conversely, the presence of specific anti-Hu CD8 T-cells has been demonstrated in PNS patients with anti-Hu Ab, suggesting a cell-mediated pathogenesis [16]. Nevertheless, anti-Hu Ab are reliable markers of cancer, especially of small-cell lung cancer (SCLC), the latter being present in more than 70% of the patients [14]. However, non-paraneoplastic cases have been reported in children and adolescents, suggesting that anti-Hu autoimmunity may also appear as a pure dysimmune condition [17]. In addition, anti-Hu Ab may be detected in 15% of neurologically-asymptomatic patients with SCLC [18]. It is likely that the development of anti-Hu response and its phenotypic expression are related to the patients’ genetic background.

Initially, two small studies (n = 17 and n = 5 patients) with incomplete HLA genotyping did not find any association between HLA and PNS with anti-Hu Ab [19, 20]. Later, an association with HLA B*7 supertype, which includes several different HLA-B alleles with overlapping peptide binding specificities [21], was reported in a small series of seven patients; along with CD8 T-cell reactivity against Hu peptides in only three patients [22]. These results were in agreement with the previously mentioned pathogenesis mediated by CD8 T-cells. More recently, it has been shown that class II HLA DQB1*02 and DRB1*03 are more frequent among anti-Hu PNS patients of Caucasian origin (62 and 47% of 53 patients studied) than in healthy, blood donors, controls (37% carried DQB1*02, p < 0.005; and 25% DRB1*03, p < 0.01); carrier frequencies were lower in neurologically asymptomatic SCLC patients lacking anti-Hu Ab (29% carried DQB1*02 and 21% DRB1*03), but did not reach significance, likely due to small sample size (n = 26) [23]. These findings suggest that CD4 T-cells may also play a role in anti-Hu PNS, and show how it is possible that different immunogenetic profiles in patients with SCLC may be associated with the presence or not of PNS. It is likely that the presentation by specific HLA molecules of certain antigens derived from tumor cells is involved in the pathogenesis of PNS. The interaction between Hu peptides and HLA should therefore be further investigated, along with those between HLA and TCR of Hu-reactive T-cells.

### Paraneoplastic cerebellar degeneration with anti-Yo autoantibodies

The second most common onconeural Ab are anti-Yo Ab, which appear in paraneoplastic cerebellar degeneration (PCD) in women with breast or ovarian cancers [13, 24]. Its pathogenesis is thought to be mediated mainly by CD8 T-cells, as Yo antigens (CDR2 and mainly CDR2L) are cytoplasmic proteins of Purkinje cells [25]. Nevertheless, humoral response seems to be also important due to the abundance of B-cells in the inflammatory infiltrates of tumors [26]. Even though Yo antigens are widely expressed in ovarian cancers, it has been shown that only those accompanied by PCD carry several genetic alterations in CDR2 and/or CDR2L loci [26].

The first studies investigating HLA in PCD with anti-Yo Ab reinforced the cell-mediated pathogenesis hypothesis, describing an association with A*24, and cytotoxic T-cell reactivity against Yo-derived peptides in A*24 carriers [27, 28]. Nevertheless, only class I genotyping was performed and sample size was small (n = 9). Later, it was found that the strongest HLA associations included class II alleles, suggesting a major role of CD4 T-cells; among 43 patients of Caucasian-origin with PCD, DQA1*03:03-DRB1*04:01 showed a protective effect (0% vs. less than 20% in control group; p < 0.05, OR = 0), but no risk haplotype was found to be shared by all patients [29]. Conversely, HLA profile in PCD depended on the associated tumor: in ovarian cancer, DQA1*01:03-DQB1*06:03-DRB1*13:01 class II haplotype was present in 33% of the patients vs. 9% of the control group (p < 0.05, OR = 8.87). This association was absent in patients with PCD and breast cancer [29]. The complex associations between HLA and PCD with anti-Yo Ab reinforce the hypothesis of involvement of both cellular and humoral immune responses. It is likely that multiple epitopes of Yo antigen or other unknown proteins are relevant in this context and are specific to the underlying cancer (ovarian or breast). Further studies are, however, necessary to investigate the HLA profiles of patients with PCD and anti-Yo Ab in larger samples, including all types of associated tumors, taking into account their histological characteristics; and, as in PNS with anti-Hu Ab, to analyze the interactions between HLA, Yo-peptides, and TCR.

### Lambert-Eaton myasthenic syndrome

Lambert-Eaton myasthenic syndrome (LEMS) is a neuromuscular junction disorder that can be either paraneoplastic or not (approximately 50%). The most common associated cancer is SCLC. Clinical presentation is similar for the two forms, with the classical triad of proximal weakness, areflexia and dysautonomia, and clinical and electromyographic post-exercise facilitation. Autoantibodies against P/Q-type voltage-gated calcium channels (anti-VGCC Ab) are present in nearly 90% of all LEMS cases, and their pathogenic role is proven. SCLC cells can express VGCC, but anti-VGCC Ab are not useful to distinguish paraneoplastic from idiopathic cases [30, 31]. In contrast, anti-glial nuclear Ab (AGNA or anti-SOX1 Ab) are strongly associated with SCLC and can be used to suspect an underlying cancer [32]. In addition, HLA differences between paraneoplastic and idiopathic LEMS have been reported in a few studies [33–37]; the largest among these included 26 paraneoplastic and 51 idiopathic patients [37]. The extended, highly conserved, haplotype 8.1 (A*01:01-B*08:01-DQB1*02:01-DRB1*03:01) or its components have been found to be more frequent in non-paraneoplastic LEMS of Caucasian-origin than in healthy controls or paraneoplastic LEMS [33–37]. HLA-B*08:01 was the locus with the strongest association: in the largest series with complete genotyping, it was carried by 69% of non-paraneoplastic LEMS vs. 23% of the control group (p < 0.001), and 12% of paraneoplastic LEMS (p < 0.001) [35, 37]. Moreover, non-paraneoplastic LEMS cases involved more frequently younger patients, commonly women, non-smokers, and with coexisting autoimmune diseases [35]. It has been also suggested that HLA-B*08:01 could improve the immunosurveillance against SCLC, as only 21% of LEMS smokers carrying this allele developed SCLC compared to 69% of non-carriers (p < 0.005, OR = 0.16, 95% CI [0.04–0.62]) [37].

In conclusion, two different pathogenic pathways may lead to LEMS: one HLA-dependent, which is likely a common substrate of other autoimmune diseases; and the other triggered by the expression of VGCC in SCLC. Nevertheless, more studies with a greater number of patients are needed to confirm this hypothesis, including different ethnic origins, and taking into account the presence or not of anti-VGCC and other Ab.

### Myasthenia gravis

Myasthenia gravis (MG) is an immune-mediated and highly heterogeneous neuromuscular junction disorder. Characteristic fatigable weakness may involve only the oculo-motor system (pure ocular MG), but may also involve axial and proximal limb muscles (generalized MG). Anti-acetylcholine receptor Ab (anti-AchR Ab) is the most frequent accompanying Ab (80% of generalized MG and 50% of ocular MG), although other Ab against proteins located at the postsynaptic membrane of the neuromuscular junction have been described. Among them, Ab against muscle-specific tyrosine kinase (anti-MusK Ab) are the most common, accounting for 40–70% of anti-AchR Ab-negative patients. In contrast to anti-AchR Ab, which are mainly of IgG1 isotype, anti-Musk Ab are IgG4, so they cannot fix complement nor bind Ig Fc domain receptors [38]. Although MG is not usually included in most PNS studies, a malignant thymoma is diagnosed in nearly 10–15% of patients, and in such cases it is almost always generalized and associated with anti-AchR Ab [39]. Furthermore, non-thymomatous MG is divided into early-onset MG (EOMG) and late-onset MG (LOMG). The age boundary between the two is usually set at 50 years, but it is not uniformly accepted and other authors have also recognized an intermediate-onset MG, between 40 and 60 years of age [38, 40]. EOMG appears typically in women, with anti-AchR Ab, and thymus hyperplasia; LOMG is more common in men, rates of anti-AchR Ab are lower, and thymus is normal or atrophic [38]. Juvenile-onset MG (JOMG) is usually defined by a MG diagnosis in patients younger than 18 years of age and it is usually a pure ocular form without anti-AchR Ab. JOMG is more frequent in East Asia than in Caucasian populations [41, 42]. Finally, anti-MusK MG is characterized by prominent bulbar involvement, poor response to usual treatments, and lack of associated thymus pathology [38].

EOMG has been typically associated with the ancestral haplotype 8.1 in Caucasian populations [40, 43–47]. Among the alleles that form this haplotype, it has been proven in large series (up to nearly 600 patients) that B*08:01 is the one that is truly associated with EOMG; the others being explained by LD [40, 46, 48]. HLA-B*08:01 had an allele frequency of 33% (vs. 13% in healthy bone marrow donors, p < 0.001, OR = 3.12, 95% CI [2.3–4.2]) in one of these studies [40]. Interestingly, this finding is the same as that found in non-paraneoplastic LEMS [37]. Nevertheless, several other different weak associations have been described in non-Western European Caucasian, American, and Asian populations [49–51].

In contrast, for LOMG there are slightly more homogeneous results across populations of Caucasian and Asian-origin. HLA-DQB1*05:02 and DRB1*16 have been reported with allele frequencies of approximately 15% (vs. less than 6% in healthy controls; p < 0.005, OR ranging from 2.95 to 5.51); no carrier frequencies were provided [52, 53]. HLA-DRB1*15:01 has been described with allele frequency of 26% (vs. 13% in healthy controls; p < 0.001, OR = 2.38, 95% CI [1.66–3.40]) and carried by 42% of LOMG patients (vs. 22% of EOMG, p < 0.05; OR = 2.5, 95% CI [1.4–4.6]), in two different studies [40, 54]. JOMG has been mainly studied in East Asia. A Japanese study on 87 patients found that the main haplotypes were DQA1*03:01-DQB1*03:03-DRB1*09:01 (68% vs. 28% in the control group; p < 0.001, RR = 5.4), and DQA1*01:02-DQB1*06:04-DRB1*13:02 (45% vs. 9%; p < 0.001; RR = 8.6) [55]. HLA-DRB1*09:01 was also frequent (50% of the alleles; usually in LD with B*46:01-DQA1*01:01-DQB1*03:03) in a Chinese series of 41 patients with JOMG, but carrier frequencies were not provided and p values were uncorrected [42]. Interestingly, a Norwegian study distinguished two clinical and genetic groups in JOMG [41]. Post-pubertal patients (n = 26) had characteristics more closely related to EOMG (40.4% carrying B*08:01, and presenting anti-AchR Ab and thymus hyperplasia), while pre-pubertal patients (n = 17) were described as more typical JOMG cases (pure ocular involvement, lower rates of anti-AchR Ab positivity) and carried more frequently DRB1*04:04 (26% vs. 7.7% of post-pubertal patients; p = 0.01, OR = 3.27, 95% CI [2.00–5.36]) [41].

Anti-MusK MG shows approximately the same HLA associations through very different populations [51, 56–60]. In a meta-analysis that included 177 patients, DQB1*05-DRB1*16 (26% vs. 9% in healthy controls; OR = 4.37, p < 0.001) and DQB1*05-DRB1*14 (19% vs. 5%; OR = 3.36, p < 0.001) were the main reported haplotypes in patients with anti-MusK MG [61]. At the allele level, DQB1*05 is carried by 78% of the patients (vs. 37%; p < 0.001, OR = 5.39), while 39% carry DRB1*14 (v. 8%; p < 0.001, OR = 5.32) and 34% DRB1*16 (vs. 10%; p < 0.001, OR = 4.98). Hence, a primary DQ effect has been proposed [61].

Finally, no consistent positive association has been described between HLA and thymomatous MG [40, 44, 50, 51, 54]. Though several studies have described different associations with both class I (A*24, A*25) and class II (DRB1*10, DQA1*04, DQB1*06) HLA alleles; these results were never reproduced, statistical analyses were not corrected for multiple comparisons, histological types of the thymomas were heterogeneous, and genotyping usually was not complete [43, 47, 53, 62, 63].

In summary, HLA associations with MG are highly diverse and in order to better define particular HLA profiles, future studies should establish uniform diagnostic criteria for each subtype of MG, especially regarding age of onset and thymus pathology.

### Chronic inflammatory demyelinating polyradiculoneuropathy with anti-neurofascin autoantibodies

Chronic inflammatory demyelinating polyradiculoneuropathy (CIDP) with Ab against neurofascin (anti-NF Ab) is an uncommon type of autoimmune neuropathy. The 155 isoform is the main antigen and is located in the paranodal loops of Schwann cells. These anti-NF Ab have the peculiarity of being mostly of IgG4 isotype. CIDP with anti-NF Ab is characterized by severe distal weakness with sensory ataxia, and disabling postural and intentional tremor of likely cerebellar origin [64–66]. Clear cerebellar ataxia and central nervous system demyelinating lesions have been also reported in a few cases [65, 66], and it has been shown that anti-NF Ab from CIDP patients bind to hippocampus and cerebellum in rat brain slides [64, 66]. Response to IVIG is usually poorer than in other types of CIDP, but rituximab may be useful in these cases [67]. Interestingly, a similar pattern of treatment response is observed in anti-Musk MG (anti-Musk Ab are also mainly IgG4) compared to MG with anti-AchR Ab [68].

Due to its scarcity and recent description, only a study that included 13 patients has thus far focused on the association between HLA and CIDP with anti-NF Ab. DRB1*15 alleles (mostly DRB1*15:01 but also DRB1*15:02) were present in 77% of the patients, usually in LD with DQB1*06. In contrast, DRB1*15 was detected in only 14% of the anti-NF Ab-negative CIDP (OR = 20, 95% CI [4.03–99.13]) and 17% of the normal population (OR = 16.9, 95% CI [4.43–57.30]) [69]. In silico studies demonstrated that the repertoire of NF peptides binding to DRB1*15:01 and DRB1*15:02 highly overlapped. Thus, both alleles may present the same peptides in a very similar fashion [69]. However, although a strong association between class II HLA and CIDP with anti-NF Ab is consistent with an Ab-mediated disease, these results must be confirmed in larger series and with complete genotyping.

### Encephalitis with anti-IgLON5 autoantibodies

The disease associated with anti-IgLON5 Ab is characterized by a wide clinical spectrum, including complex sleep disorder (non-REM and REM parasomnias, obstructive sleep apnea, central hypoventilation), bulbar dysfunction, gait disturbances, dysautonomia, ocular abnormalities, movement disorders, and cognitive impairment [70–72]. It is an enigmatic condition lying between autoimmune encephalitis and neurodegenerative diseases. On the one hand, patients have IgG4 Ab against the neuronal cell adhesion molecule IgLON5, usually in both serum and CSF, and some positive responses to immunotherapy have been reported [71–75]. These findings are in line with an immune-mediated etiology. On the other hand, pathological studies have revealed tau protein deposits without inflammatory changes [70]. Thus, whether the autoimmune response leads to neurodegeneration or vice versa, is yet unknown.

A strong class II HLA association, as in other IgG4-mediated diseases, also suggests an autoimmune pathogenesis. Patients carrying DRB1*10:01 have been reported in several case reports and small series, along with 13/15 (86.6% vs. 1.6% in the general population; OR = 36, 95% CI [19.5–67.0]) and 20/35 (57.1% vs. 2.4%; p < 0.001, OR = 54.5, 95% CI [22.2–133.9]) patients in the largest series [70, 71, 73–78]. HLA DQA1*01-DQB1*05 appears in more than 90% of the patients, not always linked to DRB1*10:01. Nevertheless, in silico studies predicted strong binding between IgLON5 peptides and DRB1*10:01 but not DQ molecules [76]. Interestingly, some clinical differences related to HLA status of the patients have been described. For instance, patients lacking DRB1*10:01 often do not demonstrate anti-IgLON5 Ab in CSF and present with a PSP-like phenotype or cognitive decline without sleep disturbances [71, 76]. In contrast, carriers usually show positivity for anti-IgLON5 Ab in the CSF and display typical sleep or bulbar features [71, 76]. Therefore, it is possible that two distinct diseases are associated with anti-IgLON5 Ab, showing different immunological, clinical and genetic features. Whether the subgroup associated with HLA is a true autoimmune disease, and the one not HLA-associated represents a neurodegenerative disease, should be further analyzed in additional anatomopathological studies and evaluating the response to prompt immunotherapy.

### Limbic encephalitis with anti-leucine-rich glioma-inactivated 1 autoantibodies

Limbic encephalitis (LE) with Ab against leucine-rich glioma-inactivated 1 (anti-LGI1 Ab) is the most common non-paraneoplastic LE [79, 80]. Faciobrachial dystonic seizures are the most typical feature of this disease and usually precede cognitive decline [81]. Anti-LGI1 LE is usually a non-paraneoplastic disorder also characterized by hyponatremia and sleep disturbances [82, 83].

LGI1 is a secreted protein that forms a trans-synaptic complex with ADAM23 (a disintegrin and metalloproteinase 23) and ADAM22, which interact with presynaptic voltage-gated potassium channels (VGKC) and postsynaptic alpha-amino-3-hydroxy-5-methyl-4-isoxazolepropionic acid receptors (AMPAR), respectively [4, 5]. Anti-LGI1 Ab are of IgG4 isotype, and therefore their pathogenic effect is thought to be due to blockage of the interaction between LGI1 and its ligands [4, 5].

A strong association with DRB1*07:01 (usually with DQB1*02:02 and DRB4 in LD) was described simultaneously by two different groups in Asian-origin (n = 11) and Caucasian (n = 25) patients. This allele was carried by nearly 90% of the patients (for the Asian series, vs. 13% in healthy controls, p < 0.001, OR = 65.8, 95% CI [8.3–522.5]; for the Dutch series, vs. 19.6% in healthy controls, p < 0.001, OR = 26.37, 95% CI [8.54–81.49]) [84, 85]. Moreover, in silico studies found that DRB1*07:01 had the highest affinity with LGI1 among all class II HLA, likely binding to the leucine-rich repeat domain [84]. In contrast, though DRB1*07:01 carrier frequencies were very similar (91% of 68 patients; p < 0.001, OR = 27.6, 95% CI 12.9–72.2]), in silico results were not confirmed in a third study [86]. Similarly, while van Sonderen et al. suggested that paraneoplastic anti-LGI1 LE was not associated with DRB1*07:01 (2/4 patients with tumors did not carry this allele) [85], Binks et al. [86] did not find differences between patients with (n = 9) and without tumors (n = 59).

Other minor HLA associations reported with anti-LGI1 LE include several class I HLA alleles (B*44:03, OR, 13.9; B*57:01, OR = 3.7; C*06:02, OR = 3.9; C*07:06, OR = 26.5), but none of them have been found in more than one study [84–86]. Interestingly, B*57:01 and C*06:02, which are known to be linked respectively to antibiotic-induced rash and psoriasis, were detected in patients presenting with these comorbidities [86]. Although cutaneous reactions related to antiepileptic drugs are a major concern in anti-LGI1 LE [87], no HLA associations have been yet described in this subgroup of patients [86].

In conclusion, anti-LGI1 LE shows a strong association with DRB1*07:01, suggesting an important role of HLA-mediated antigen presentation. Nevertheless, it remains unknown whether phenotypical differences exist between carrier and non-carrier DRB1*07:01 patients, as well as which is the HLA profile in the uncommon paraneoplastic cases.

### Diseases with anti-contactin-associated protein-like 2 autoantibodies

Contactin-associated protein-like 2 (CASPR2) plays an important role in the clustering of VGKC at the juxtaparanodes in the peripheral nervous system, but it is also expressed by inhibitory neurons in the central nervous system. Anti-CASPR2 Ab are mainly of IgG4 isotype as is the case with anti-LGI1 Ab [4, 5]. They are associated with three major syndromes: LE, neuromyotonia (NMT), and Morvan’s syndrome (MoS) [88, 89]. These different clinical phenotypes seem to reflect distinct underlying pathogenetic mechanisms. For example, MoS commonly coexist with MG and malignant thymoma, a unique constellation not detectable in the other syndromes associated with anti-CASPR2 Ab [89, 90]. Moreover, NMT and MoS do not have anti-CASPR2 Ab in CSF, and MoS patients may have additionally anti-LGI1 Ab in serum [89, 90].

DRB1*11:01 has been reported in 48% of 31 patients with anti-CASPR2 Ab (vs.9% in healthy controls; p < 0.001, OR = 9.4, 95% CI [4.6–19.3]) [86]. DQA1*05:01-DQB1*03:01 were also detected due to LD with DRB1*11:01. Interestingly, the heterodimer DQA1*05:01-DQB1*03:01 was predicted in silico to bind specifically some CASPR2-derived peptides [86]. Thus, this haplotype may be involved in the presentation of CASPR2 antigens.

No difference in HLA status has been described among the three main clinical syndromes, even though the number of MoS (n = 3) and NMT (n = 2) in the study was low. Similarly, it has not been shown if paraneoplastic patients carry or not this distinct haplotype, as only four patients with tumors were included, none of them with MoS and malignant thymoma [86]. Thus, further studies are needed to clarify whether all syndromes associated with anti-CASPR2 Ab share the same HLA profile, and whether DRB1*11:01 is also more frequently found in patients of different ethnic origins.

### Encephalitis with anti-*N*-methyl-d-aspartate receptor autoantibodies

Anti-NMDAR encephalitis is the most common autoimmune encephalitis and is even more frequent than infectious etiologies [79, 80, 91]. Its clinical features are now well known, including different combinations of psychiatric symptoms, cognitive and speech dysfunction, seizures, movement disorders, decreased level of consciousness, dysautonomia, and central hypoventilation [92]. Anti-NMDAR encephalitis may be paraneoplastic, usually in association with ovarian teratomas in young women and carcinomas in elderly patients [92, 93]. In contrast, most children present with non-paraneoplastic disease [92]. Recently, herpes-simplex virus encephalitis has been identified as another potential trigger of anti-NMDAR encephalitis [94]. This variety of etiopathogenic contexts may be explained by different genetic predispositions.

Anti-NMDAR Ab are of IgG1 isotype, in contrast to anti-LGI1 and anti-CASPR2 Ab. Their pathogenic role has been proven both in vitro (leading to crosslink, internalization and finally reducing the number of NMDAR at the synapsis) and in vivo [4, 5]. No strong HLA association has been reported with anti-NMDAR encephalitis thus far [84, 95]. DRB1*16:02 was found to be more frequent in patients than in controls, but it accounted for less than 15% of the alleles of a series of 61 patients (vs. less than 5% in control group; p < 0.05, OR = 3.41, 95% CI [1.81–6.17]) [96]. Moreover, carrier frequencies between paraneoplastic (mostly ovarian teratomas) and non-paraneoplastic cases were similar. The only observed clinical difference was a possible worse response to treatment in DRB1*16:02 carriers [96]. Finally, in a genome-wide association study, a weak association with B*07:02 was described in adult patients with anti-NMDAR encephalitis (21% of the alleles of a sample of n = 49; p < 0.05, OR = 2.32, 95% CI [1.34–4.00]) [95]. It therefore seems that anti-NMDAR encephalitis have a less clear association with HLA than IgG4-mediated disorders, likely related to the different IgG-isotype involved. However, larger samples are necessary to confirm this hypothesis, and to try to identify other non-HLA loci that could be relevant in the genetic predisposition of this disease.

### Neurological syndromes with anti-glutamic-acid decarboxylase autoantibodies

Glutamic acid decarboxylase (GAD) is intracellularly located at the synapsis, and hence the pathogenic role of anti-GAD Ab is controversial [5]. Anti-GAD Ab are mainly present in three neurological syndromes: LE, stiff-person syndrome (SPS), and cerebellar ataxia (CA). Mild or partial forms of all of these have been described, such as temporal lobe epilepsy, stiff-leg syndrome or isolated nystagmus [97, 98]. Anti-GAD Ab are also present at lower titers in serum from patients with organ-specific autoimmune diseases, especially in type 1 diabetes mellitus [99]. Moreover, these organ-specific autoimmune diseases are often seen in patients and their relatives, suggesting a common genetic predisposition [97–99].

Type 1 diabetes mellitus is strongly associated with class II HLA haplotypes DQA1*05:01-DQB1*02:01-DRB1*03:01 (DQ2-DR3) and DQA1*03:01-DQB1*03:02-DRB1*04 (DQ3-DR4) [7]. DQ2-DR3 is part of the haplotype 8.1 and is also related to other autoimmune diseases, such as autoimmune thyroid disease and celiac disease [7]. Because of this, DQB1*02:01 and DRB1*03:01 were reported independently in two series of SPS, describing carrier frequencies of 44% (n = 18) and 72% (n = 18), respectively; genotyping only included class II HLA and the p values provided were uncorrected [100, 101]. DQ2-DR3 haplotype was also reported in 4/5 SPS and 2/6 CA patients [102]. In contrast, DQ3-DR4 haplotype has not been associated with neurological diseases with anti-GAD Ab. More recently, the uncommon haplotype DQA1*01:02-DQB1*05:02-DRB1*15:01 was shared by two related patients presenting with SPS and CA, suggesting that other HLA haplotypes may be related with neurological diseases with anti-GAD Ab [103].

Despite being one of the first neurological diseases with Ab in which HLA was analyzed, what we know about HLA in neurological syndromes with anti-GAD Ab remains very superficial. Larger studies including all clinical presentations and with complete genotyping, should enable to confirm whether these diseases share a common HLA profile with systemic organ-specific autoimmune diseases.

### Neuromyelitis optica

Once considered as a severe variant of multiple sclerosis (MS), neuromyelitis optica (NMO) is now defined as a well-differentiated disease thanks to the description of serum IgG1 anti-AQP4 Ab [1, 2]. Neuromyelitis optica spectrum disorders (NMOSD) include longitudinally extensive transverse myelitis and optic neuritis, along with cerebral syndromes involving area postrema, brainstem, and diencephalon [104]. NMO is not only immunological and clinically distinct from MS, but also shows a different epidemiology. NMO is more frequent in African, East Asian, and Latin American populations; while MS is more common in Caucasian ones [105]. This particular geographical distribution is obviously also accompanied by different HLA associations.

Several HLA investigations have reported an association between NMO and DRB1*03:01 in Caucasian or mixed-origin patients [105–111], although these results are not consistently found [112, 113]. One limitation of most of these studies was the inclusion of anti-AQP4 Ab positive and negative patients, as well as NMO and NMOSD. Nevertheless, several sub-analyses, and a recent study including 132 patients with NMO and anti-AQP4 Ab, showed that the DRB1*03:01 association depended on anti-AQP4 Ab positivity (p < 0.001, OR = 4.09, 95% CI [2.91–5.74]) [106, 109, 110, 114]. Another minor association described in the same populations is DRB1*10:01, but the p values provided were uncorrected [105, 107, 109–111].

Associations between HLA and NMO in patients from East Asia were initially even more difficult to interpret, partially due to the analysis of opticospinal MS (now considered as a synonym of NMO) along with “conventional” MS [115, 116]. Nowadays, the most important alleles that have been specifically linked to anti-AQP4 Ab positivity are DRB1*16:02 (carried by 9% vs. 0.8% in healthy controls; p < 0.01), and DPB1*05:01 (85% vs. nearly 65% in healthy controls; p < 0.01) [116–118]. A negative association has been also described with DRB1*09:01 (6% vs. 27.5% in healthy controls; p < 0.01, OR = 0.16, 95% CI 0.07–0.37), regardless of the anti-AQP4 Ab status [118]. Interestingly, a recent familial case report from Taiwan described a woman with NMO who was heterozygote for DRB1*03:01/16:02 and who transmitted DRB1*03:01 to her NMO-affected daughter, who also carried DPB1*05:01 inherited from her father [119]. This example reflects the complex interactions among risky HLA alleles at the same or different loci.

Thus, two major associations have been described between HLA and NMO patients depending on the population origin, and in all cases the association seems to be more evident for anti-AQP4 Ab positive cases. Recently, it has been shown that some patients with reportedly seronegative NMO present Ab against myelin oligodencrocyte glycoprotein (MOG), configuring a distinct disease [120, 121]. The HLA profile of anti-MOG disease remains unknown, but it is likely to be different from the one of anti-AQP4 NMO. Future studies on HLA associations with demyelinating diseases should rely on well-defined clinical phenotypes accompanied by specific biomarkers (ideally Ab) in order to perform genotyping on uniform populations. This will allow us to obtain more consistent results that may lead to a better understanding of the etiopathogenesis of these diseases.

## Conclusions

Description of new Ab has enabled to establish more accurate diagnosis and to expand our knowledge about autoimmune neurological diseases. However, the primary trigger that promotes the production of these Ab and the activation of other immune response effectors is uncertain. It is likely that a complex interaction between environmental factors and genetic background may explain why some individuals develop autoimmune diseases. HLA is a cornerstone within the immune system and it has been already associated with many non-neurological autoimmune disorders. Interestingly, several autoimmune neurological diseases with Ab share two common aspects regarding their HLA associations. First, IgG4-mediated neurological diseases show a strong link to specific class II HLA haplotypes (Table 1), as it has been also shown in other uncommon non-neurological entities with IgG4 [122]. Conversely, disorders with Ab of IgG1 isotype are mainly related to the ancestral haplotype 8.1 (Table 2), which is known to be associated with many other systemic autoimmune diseases, such as type 1 diabetes mellitus or autoimmune thyroid disease. Second, non-paraneoplastic diseases seem to be more homogeneous in their HLA status, maybe reflecting that abnormalities in peptide presentation are critical in the pathogenesis in a non-tumor context, while cancer is a sufficiently powerful immune trigger irrespective of the patient’s genotype. More studies are needed, including subjects of all ethnic origin and analysis of peptide-binding properties in order to better define the role of HLA in autoimmune neurological diseases.

**Table 1:** Main HLA associations with IgG4-mediated neurological diseases

Autoantibody	HLA	Carriers	References
Anti-Musk	DQB1*05	78%	[61]
	DQB1*05-DRB1*14	26%	[61]
	DQB1*05-DRB1*16	20%	[61]
Anti-NF	DRB1*15	77%	[69]
Anti-IgLON5	DQB1*05:01-DRB1*10:01	57–90%	[70, 71, 76]
	DQA1*01-DQB1*05	≈ 90%	[76]
Anti-LGI1	DRB1*07:01	≈ 90%	[84–86]
Anti-CASPR2	DRB1*11:01	48%	[86]

*CASPR2* Contactin-associated protein-like 2, *HLA* Human leukocyte antigen, *LGI1* leucine-rich glioma-inactivated 1, *MusK* muscle-specific tyrosine kinase, *NF* neurofascin 155

**Table 2:** Main HLA associations with neurological diseases with autoantibodies of IgG1 isotype

Autoantibody	HLA	Population	References
Anti-Hu	DQB1*02-DRB1*03	SCLC-PNS	[23]
Anti-Yo	DQA1*01:03-DQB1*06:03-DRB1*13:01	Ovarian cancer	[29]
Anti-VGCC	B*08:01	Non-paraneoplastic LEMS (69%)	[37]
Anti-AchR	B*08:01	EOMG	[40, 46, 48]
	DQB1*05-DRB1*16	LOMG	[52, 53]
	DRB1*15	LOMG	[40, 54]
	DQA1*03-DQB1*03-DRB1*09	EOMG	[55]
	DQA1*03-DQB1*06-DRB1*13	EOMG	[55]
	DRB1*04:04	Pre-pubertal EOMG	[41]
Anti-NMDAR	DRB1*16:02		[96]
	B*07:02	Adult onset	[95]
Anti-GAD	DQB1*02-DRB1*03	SPS & CA	[100–102]
Anti-AQP4	DRB1*03	Caucasian and mixed	[106, 109, 110, 114]
	DPB1*05:01	East Asia	[117, 118]
	DRB1*16:02	East Asia	[117, 118]

*AchR* acetylcholine receptor, *AQP4* aquaporin 4, *CA* cerebellar ataxia, *EOMG* early-onset myasthenia gravis, *GAD* glutamic acid decarboxylase, *HLA* human leukocyte antigen, *JOMG* juvenile-onset myasthenia gravis, *LEMS* Lambert-Eaton myasthenic syndrome, *LOMG* late-onset myasthenia gravis, *NMDAR N*-methyl-d-aspartate receptor, *PNS* paraneoplastic neurological syndrome, *SCLC* small-cell lung cancer, *SPS* stiff-person syndrome, *VGCC* voltage-gated calcium channels

## Data Availability

Not applicable.
